# ﻿First report of the genus *Woonpaikia* Park, 2010 (Lepidoptera, Lecithoceridae) from China, with the description of two new species

**DOI:** 10.3897/zookeys.1192.115033

**Published:** 2024-02-19

**Authors:** Shuai Yu, Shuxia Wang

**Affiliations:** 1 College of Life Sciences, Liaocheng University, Liaocheng 252000, China Liaocheng University Liaocheng China; 2 College of Life Sciences, Nankai University, Tianjin 300071, China Nankai University Tianjin China

**Keywords:** Gelechioidea, Lecithocerinae, new record

## Abstract

The lecithocerid genus *Woonpaikia* Park, 2010 and *Woonpaikiaangoonae* Park, 2010 are newly recorded from China. *Woonpaikiasimilangoonae* Yu & Wang, **sp. nov.** and *W.imperspicua* Yu & Wang, **sp. nov.** are described as new to science. Images of adults of the Chinese *Woonpaikia* species are provided, along with a key to the males of all the known species of *Woonpaikia*.

## ﻿Introduction

*Woonpaikia* Park, 2010 is a small genus of lepidopteran classified in the family Lecithoceridae, subfamily Lecithocerinae. Park (2010) erected the genus to accommodate *W.villosa* and *W.angoonae* from Thailand, with *W.villosa* as the type species. Since then, no more species have been described. *Woonpaikia* is morphologically similar to the type genus of Lecithoceridae, *Lecithocera* Herrich-Schäffer, 1853, in sharing a similar wing pattern and venation, but it can be distinguished by presence of a ventrodistal pectin-like scale tuft on the scape of the antennae, labial palpi dorsally with dense, long, hair-like scales, male genitalia with the juxta decrescent and the sacculus produced apically to form a process.

Here we describe two new species of *Woonpaikia*. We also provide new distribution records for other known species in China, as well as a key to identify males of all the known species of this genus.

## ﻿Materials and methods

The specimens examined were collected in China using 450 W high-pressure mercury lamps. Morphological terminology in the descriptions follows Gozmány (1978). Wingspan was measured from the tips of the left to right forewings. Slides of genitalia were prepared following the methods introduced by Li (2002). Photographs of the adults were taken with a Leica M205A stereomicroscope, and photographs of genitalia were taken with a Leica DM750 microscope plus the Leica Application Suite v. 4.6. All photographs were refined with Photoshop CC.

Materials examined, including the type series of the new species, are deposited in Liaocheng University, Liaocheng, China (**LCU**), except for several specimens of *W.angoonae*, which are deposited in the Insect Collection of Nankai University, Tianjin, China (**NKU**).

## ﻿Taxonomic accounts

### ﻿ Woonpaikia

Taxon classificationAnimaliaLepidopteraLecithoceridae

Park, 2010

B102BAF5-C003-5EE4-B6C7-F9B15CEDE6B6

Woonpaikia Park, 2010: 239. Type species: Woonpaikiavillosa Park, 2010.

### ﻿Key to the males of *Woonpaikia*

**Table:** 

1	Apical process of sacculus extending posteriorly at least as far as apex of cucullus (Fig. 3A, C)	**2**
–	Apical process of sacculus extending much less than length of cucullus (as in Fig. 3B)	**3**
2	Cucullus capitate; aedeagus with a needle-like apical extension (Fig. 3C)	***W.similangoonae* sp. nov.**
–	Cucullus acuminate; aedeagus without apical extension (Fig. 3A)	***W.angoonae***
3	Apical process of sacculus triangular; width of cucullus at middle about twice width at base (Fig. 3B)	***W.imperspicua* sp. nov.**
–	Apical process of sacculus horn-shaped; width of cucullus at only middle slightly greater than at base (Park 2010: 241, fig. 10)	***W.villosa***

### ﻿ Woonpaikia angoonae

Taxon classificationAnimaliaLepidopteraLecithoceridae

Park, 2010

5EA3C448-CC7C-58DB-848B-014045AE2E86

Figs 1A–C , 3A

Woonpaikia angoonae Park, 2010: 241. Holotype male collected in Thailand (Chiang Mai) deposited in Osaka Prefecture University, Osaka, Japan (OPU).

#### Materials examined.

China • 1♂; Yunnan, Mengla County, Bubeng; 21.60°N, 101.60°E; 652 m elev.; 14 July 2013; SR Li et al. leg.; slide no. YS19297, NKU • 1♂; Yunnan, Xishuangbanna, Yexianggu; 22.17°N, 100.87°E; 762 m elev.; 9 July 2015; KJ Teng & X Bai leg.; slide no. YS19298, NKU • 1♂; Yunnan, Jinghong; 21.90°N, 100.77°E; 640 m elev.; 2 Aug. 2016; KJ Teng et al.; slide no. YS19293, NKU • 1♂; Yunnan, Menghai County, Nabanhe; 22.25°N, 100.61°E; 1210 m elev.; 4–5 Aug. 2022; S Yu & KJ Teng leg.; slide no. YUS061, LCU • 3♂; Yunnan, Jinghong, Mt Jinuo; 21.98°N, 100.89°E; 1425 m elev.; 6–7 Aug. 2022; S Yu & KJ Teng leg.; slide no. YUS063, LCU.

#### Description.

**Figure 1.**
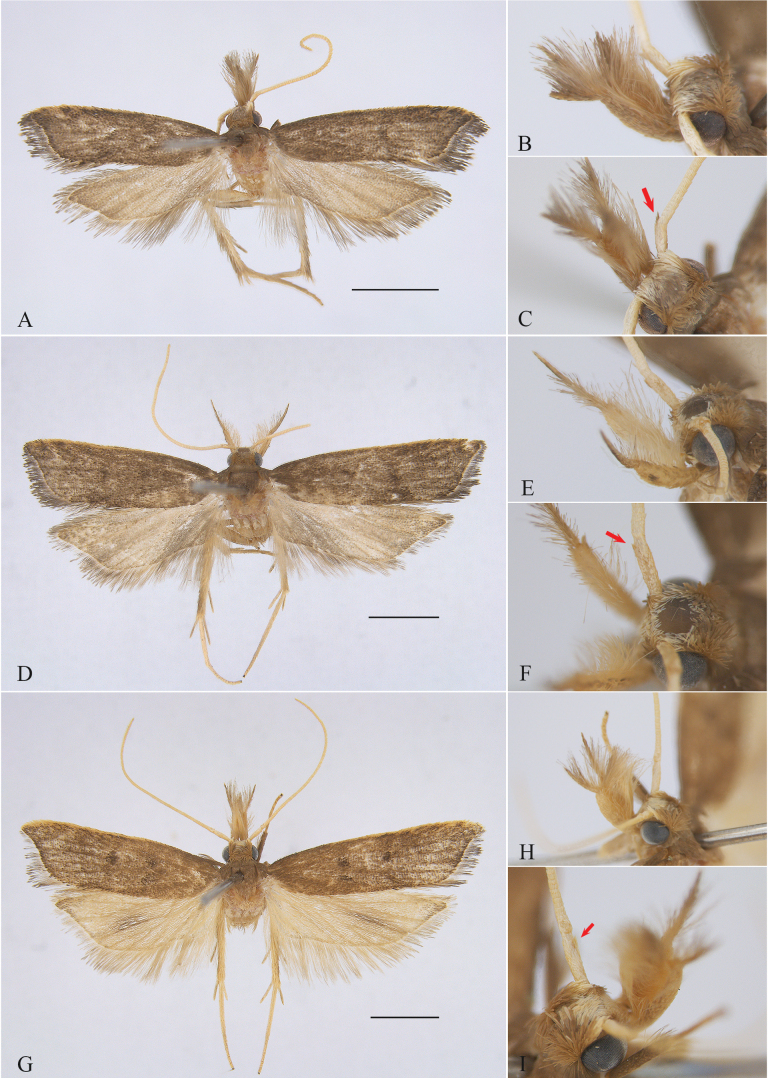
External features of *Woonpaikia* spp. **A–C***W.angoonae* Park, 2010, male, YUS063 **B** lateral view of head **C** close-up of scape **D–F***W.imperspicua* sp. nov., holotype, male, YUS064 **E** lateral view of head **F** close-up of scape **G–I***W.similangoonae* sp. nov., male, YUS062 **H** lateral view of head **I** close-up of scape. Scale bars: 2.0 mm.

Adult wingspan 10.5‒12.5 mm (Fig. 1A).

#### Diagnosis.

This species can be recognized by the smoothly arcuate apical process of the sacculus which extends posteriorly beyond the apex of the cucullus (Fig. 3A). It is most similar to the new species, *W.similangoonae*. The differences between these species are detailed below.

#### Distribution.

China (Yunnan, new record), Thailand.

#### Remarks.

This species was originally described from Thailand based on a single male. It is recorded here from China for the first time.

### ﻿ Woonpaikia imperspicua

Taxon classificationAnimaliaLepidopteraLecithoceridae

Yu & Wang sp. nov.

4CA60D61-019A-5AEC-8B2C-48807F16527B

https://zoobank.org/90F5CE81-246C-4C58-9D51-A37832D89FF0

Figs 1D–F , 2A , 3B

#### Type materials.

***Holotype***: China • ♂; Yunnan, Jinghong, Mt Jinuo; 21.98°N, 100.89°E; 1425 m elev.; 6 Aug. 2022; S Yu & KJ Teng leg.; slide no. YUS064, LCU. ***Paratype***: 1♂; same data as holotype; slide no. YUS060, LCU.

#### Diagnosis.

The new species can be distinguished by the triangular apical process of the sacculus which extends for less than 1/2 the length of the cucullus, and by the aedeagus which has dorsal and ventral extensions at the apex; in *W.similangoonae* and *W.angoonae*, the apical process of the sacculus is long, extending posteriorly at least as far as the apex of the cucullus (sometimes further). *Woonpaikiavillosa* has a transverse fascia in the hindwing (Park 2010: 240, fig. 1), whereas *W.imperspicua* lacks this fascia.

#### Description.

**Figure 2.**
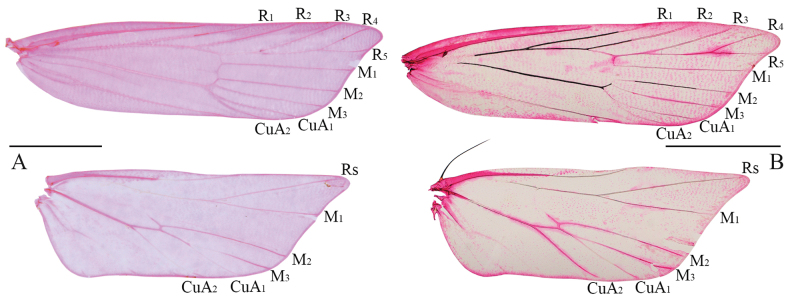
Wing venation of *Woonpaikia* spp. **A***W.imperspicua* sp. nov., paratype, slide no. YUS060 **B***W.similangoonae* sp. nov. paratype, slide no. YUS075. Scale bars: 2.0 mm.

Wingspan 13.5‒14.0 mm (Figs 1D, 2A). Head brown. Antenna orange white; scape with a small imperceptible pectin-like scale tuft ventrodistally. Labial palpus dorsally with dense, long, hair-like scales; third palpomere shorter than second palpomere. Forewing slightly widened distally, costal margin almost straight, apex produced, termen gently concave; ground color dark brown; orange-yellow along costal margin from before middle to apex; discal stigma black, small, rounded; plical stigma black; discocellular stigma black, larger than plical stigma; fringe greyish brown, with an orange-white basal line; R_3_, R_4_ and R_5_ stalked, R_5_ to termen, CuA_1_ and CuA_2_ with short stalk. Hindwing and fringe pale greyish brown; M_3_ and CuA_1_ stalked.

**Figure 3.**
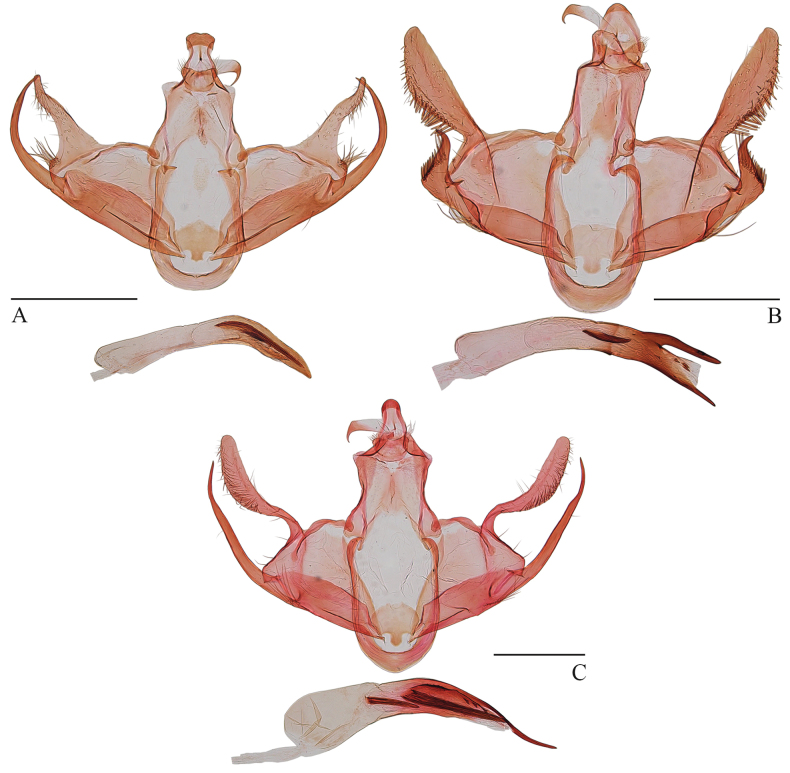
Male genitalia of *Woonpaikia* spp. **A***W.angoonae* Park, 2010, slide no. YUS063 **B***W.imperspicua* sp. nov., holotype, slide no. YUS064 **C***W.similangoonae* sp. nov., slide no. YUS062. Scale bars: 0.5 mm.

***Male genitalia*** (Fig. 3B). Uncus with caudal lobes thumb-shaped. Gnathos with basal plate distally semi-ovate, with rounded apex; median process almost uniformly wide in basal 2/3, thereafter sharply narrowed to a pointed apex, curved ventrad at basal 2/3 by a right angle. Costal bar narrow, taenioid. Valva with basal part subquadrate; cucullus arising from upper corner of basal part of valva, narrowed at base, widened to middle, width at middle about twice width of base, thereafter narrowed to blunt apex, nearly straight on costal margin, bearing a row of needle-like setae along ventral margin; sacculus wide, straight on its ventral margin, with a triangular apical process extending less than 1/2 length of cucullus and bearing a row of needle-like setae. Juxta elliptical, wider than long, with a subquadrate process at middle on anterior margin. Vinculum rounded on anterior margin. Aedeagus slightly shorter than valva, almost uniformly wide, with a horn-like dorsal extension and a spiniform ventral extension; cornuti consisting of a flake-like plate placed beyond middle and three spinules near apex.

**Female.** Unknown.

#### Distribution.

China (Yunnan).

#### Etymology.

The specific name is derived from the Latin *imperspicuus*, referring to the small imperceptible pectin-like scale tuft on the scape of the antenna.

### ﻿ Woonpaikia similangoonae

Taxon classificationAnimaliaLepidopteraLecithoceridae

Yu & Wang sp. nov.

1F83182E-372E-5AB9-BD7A-6128DD9BA4E1

https://zoobank.org/039D1A18-1348-4893-A6F8-4B5D5A086F33

Figs 1G–I , 2B , 3C

#### Type materials.

***Holotype***: China • ♂; Yunnan, Mang City, Mt Banggunjian; 24.39°N, 97.84°E; 1758 m elev.; 14 Aug. 2022; S Yu & KJ Teng leg.; slide no. YUS062, LCU. ***Paratype***: 1♂; same data as holotype; slide no. YUS075, LCU.

#### Diagnosis.

The new species is similar to *W.angoonae*, but it can be distinguished by the capitate cucullus and the aedeagus with a needle-like apical extension. In *W.angoonae*, the cucullus is acuminate and the aedeagus lacks an extension on the apex.

#### Description.

Wingspan 14.0‒15.0 mm (Figs 1G, 2B). Head yellowish brown. Antenna yellow; scape ventrodistally with a small imperceptible pectin-like scale tuft. Labial palpus with dense, long, hair-like scales dorsally; third palpomere shorter than second palpomere. Forewing slightly widened distally, with costal margin almost straight, apex produced, termen gently concave; ground color yellowish brown; orange-yellow along costal margin from about basal 2/5 to apex; discal stigma black, rounded; plical stigma black, nearly same size as discal stigma; discocellular stigma black, elliptical; fringe greyish brown, with an orange-white basal line; R_3_, R_4_, and R_5_ stalked, R_5_ to termen, CuA_1_ and CuA_2_ with short stalk. Hindwing and fringe yellow, dark brown scales along vein M_2_; M_3_ and CuA_1_ shortly stalked.

***Male genitalia*** (Fig. 3C). Uncus nearly inverted trapezoidal; caudal lobes semi-ovate. Gnathos with basal plate roundly produced on posterior margin; median process narrowed slightly from base to basal 2/3, thereafter sharply narrowed to a pointed apex, curved ventrad at basal 2/3 by a right angle. Costal bar narrow, arched taenioid. Valva with basal part trapezoidal; cucullus capitate, arising from upper corner of basal part of valva, sinuate, narrow basally, widened to about basal 2/3, thereafter narrowed slightly to rounded apex, costal margin arched in basal 1/2 and straight in distal 1/2; sacculus wide, straight on its ventral margin, with a long, apical process extending posteriorly as far as apex of cucullus. Juxta elliptical, wider than long, with a thumbed process at middle on anterior margin. Vinculum subrounded on anterior margin. Aedeagus slightly shorter than valva, wide at base, narrowed to apex, with a needle-like apical extension; cornuti consisting of two needle-like spines of different sizes and a flake-like plate bearing three spinules.

**Female.** Unknown.

#### Distribution.

China (Yunnan).

#### Etymology.

The specific epithet is derived from the Latin *simile* (likeness) and *angoonae*, referring to the similarity between this new species and *W.angoonae*.

## Supplementary Material

XML Treatment for Woonpaikia

XML Treatment for Woonpaikia angoonae

XML Treatment for Woonpaikia imperspicua

XML Treatment for Woonpaikia similangoonae

## ﻿Additional information

### Author ORCIDs

Shuai Yu https://orcid.org/0000-0003-3670-2701

Shuxia Wang https://orcid.org/0000-0002-9316-6661

### Data availability

All of the data that support the findings of this study are available in the main text.
